# Gait Characteristics Based on Shoe-Type Inertial Measurement Units in Healthy Young Adults during Treadmill Walking

**DOI:** 10.3390/s20072095

**Published:** 2020-04-08

**Authors:** Myeounggon Lee, Changhong Youm, Byungjoo Noh, Hwayoung Park

**Affiliations:** 1 Biomechanics Laboratory, College of Health Sciences, Dong-A University, Saha-gu, Busan 49315, Korea; ssam011@dau.ac.kr (M.L.); app00113@dau.ac.kr (H.P.); 2 Department of Healthcare and Science, College of Health Sciences, Dong-A University, Saha-gu, Busan 49315, Korea; bnoh@dau.ac.kr

**Keywords:** inertial measurement units, wearable, variability, walking, sensor, normative values

## Abstract

This study investigated the gait characteristics of healthy young adults using shoe-type inertial measurement units (IMU) during treadmill walking. A total of 1478 participants were tested. Principal component analyses (PCA) were conducted to determine which principal components (PC_s_) best defined the characteristics of healthy young adults. A non-hierarchical cluster analysis was conducted to evaluate the essential gait ability, according to the results of the PC_1_ score. One-way repeated analysis of variance with the Bonferroni correction was used to compare gait performances in the cluster groups. PCA outcomes indicated 76.9% variance for PC_1_–PC_6_, where PC_1_ (gait variability (GV): 18.5%), PC_2_ (pace: 17.8%), PC_3_ (rhythm and phase: 13.9%), and PC_4_ (bilateral coordination: 11.2%) were the gait-related factors. All of the pace, rhythm, GV, and variables for bilateral coordination classified the gait ability in the cluster groups. We suggest that the treadmill walking task may be reliable to evaluate the gait performances, which may provide insight into understanding the decline of gait ability. The presented results are considered meaningful for understanding the gait patterns of healthy adults and may prove useful as reference outcomes for future gait analyses.

## 1. Introduction

Gait analysis is used to evaluate the physical function, health status, and quality of life [1,2]. It is also employed to identify pathology and evaluate the progression of diseases [3]. Gait analysis has been conducted to investigate the effect of gait disturbances on healthy humans and patients [3]. For instance, the walking speed is a variable commonly used for determining the ability to walk, whereby decreased walking speeds are anticipated in the cases of aging and disease [1,2,3,4,5,6]. Especially, slower walking speed and shorter stride length indicate weakness of the lower limbs [2,4]. In addition, gait variability (GV) and bilateral coordination are variables that are used to evaluate the dynamic stability during gait [7,8,9]. Greater GV and bilateral coordination values during gait reflect poor gait stability. These symptoms may suggest weaker muscles and decline motor function, which affect the gait automaticity [1,2,7,10,11,12,13]. 

Several gait analysis studies have been conducted in children [14], young adults [15], the elderly [1,6], and in all normal volunteers in other age ranges [3,5]. Some studies suggested the need to conduct gait analyses to determine the dependence of gait on age, race, and sex [2,14,15]. However, most previous studies conducted over-ground walking tests in straight walkways that spanned 6 m with the use of two to four steps per trial. Accordingly, multiple trials had been performed to collect data using multiple steps. However, it is uncertain if the methods based on repeated and averaged patterns, which do not use continuous walking steps and yield outcomes comparable to the actual walking patterns in individuals because such methods may have difficulty in mimicking real walking patterns [16].

By contrast, walking on treadmills may be more useful for the collection of data pertaining to numerous continuous walking steps in comparison with methods involving repeated and averaged patterns due to decreased space requirements, multiple repetitions of strides, and controllability of the walking speed [17]. Recently, many studies have reported the positive effects of gait training programs using treadmills on amputees [18] and pathological patients such as people suffering from cerebral palsy [19], Parkinson’s disease [20,21,22,23,24,25], and spinal cord injury [26]. However, several studies reported limitations such as lack of controls [19,24,25] and relatively small samples of patients [20,22]. Thus, establishing normative values based on treadmill walking may be useful in a clinical environment as reference values [1,3,6]. Therefore, gait analyses conducted on healthy human volunteers, who had experiences in using the treadmill, have been based on multiple continuous steps and have provided reliable and objective data. The determination of the gait characteristics of healthy young adults may provide important knowledge for understanding the gait patterns of healthy individuals and for patients with movement disorders.

In addition, utilizing a shoe-type inertial measurement unit (IMU) system has advantages when compared with previous studies using IMU sensors. The IMU sensors are mounted on both the left and right outsoles, which may enhance their position stability as well as decrease the motion artifacts and dynamic acceleration in comparison with the case where the IMU sensor is placed on the lumbar vertebra [27,28], lateral malleolus [29], or shoe [30]. Furthermore, the foot is the initial segment that contacts the ground during walking. Thus, the shoe-type IMU system may be an effective and objective technique to detect gait events, which may enhance the data accuracy [31,32,33]. Another system that detects the gait events by foot pressure is the smart socks system. One of the major advantages of this system is that it is capable of enhancing the accuracy of the gait events [34], as in the shoe-type IMU system. 

Therefore, the purpose of this study was the investigation of the gait characteristics in healthy young adults using shoe-type IMU during treadmill walking (one-minute intervals). The primary purpose was to examine which variables best defined the characteristics of healthy young adults with principal component analysis (PCA). The secondary purpose was to establish the normative reference values for the principal components (PC_s_) such as the spatiotemporal parameters (pace, rhythm, and phases), GV, and the variables for bilateral coordination in all the participants as well as male and female. Lastly, the third purpose was to determine the characteristics of gait performance based on the clustered groups, according to the results of the PC_1_ score. Thus, we classified the clustered groups to determine the respective gait characteristics of young adults. We hypothesized that the participants who had altered gait variables indicated poorer gait performance based on the gait-related variables than the other participants.

## 2. Methods

### 2.1. Study Population

Participants were recruited as part of a community-wide survey in the Busan metropolitan city from February to December 2017. We contacted 1600 participants aged between 19 and 30 living in the community, and recruited 1514 young adults for this study (response rate: 94.6%). None of the participants had any history of musculoskeletal or neurological problems that affected gait, and all participants were able to walk without any support. Thirty-six participants were excluded from the study because they did not complete the testing for personal reasons. In total, 1478 participants (males: 558, females: 920) successfully performed the treadmill walking test (response rate: 92.4%) (Table 1). All the participants read and signed an informed consent form that was approved by the Institutional Review Board of Dong-A University (IRB number: 2–104709–AB–N–01–201709–HR–045–02).

### 2.2. Instrumentation

Shoe-type IMU systems (DynaStab^TM^, JEIOS, South Korea), which consisted of shoe-type data loggers (Smart Balance SB-1^®^, JEIOS, South Korea) and a data acquisition system, were utilized in this study. The shoe-type data logger included an IMU sensor (IMU–3000^TM^, InvenSense, USA) that could measure triaxial acceleration (up to ± 6 g) and tri-axial angular velocities (up to ± 500° s^−1^) along the three orthogonal axes [31,33]. The IMU sensors were installed on the outsoles of both shoes, and the data were transmitted wirelessly to a data acquisition system via Bluetooth^®^. The shoe-type IMU system showed excellent data agreement of the spatiotemporal parameters when compared with a three-dimensional motion capture system for healthy adults and patients with Parkinson’s disease (intra-class coefficient value: 0.97–0.99) [31,33]. The shoe sizes were adapted to fit the tested individuals, and a range of shoe sizes was available (from 225 to 280 mm). The speed of the belt on the treadmill (HK–365, Healthkeeper, South Korea) could be controlled from 0.5 to 16 km/h in increments of 0.1 km/h.

### 2.3. Test Procedures

Prior to the treadmill walking test, biometric data were recorded for all the participants, including body height, weight, and body fat percentage. All participants completed questionnaires to assess their responses to physical activity (PA). PAs were evaluated with the use of the international PA questionnaire–short form (IPAQ–SF), which was composed of seven items that pertained to the self-reported PA of the participants, such as high, moderate, and walking PA. Based on these questionnaires, we assessed the frequency (days/week) and calculated the metabolic equivalents (Metabolic equivalents (METs)/week) [35]. 

All the participants performed over-ground walking tests in a straight 10 m walkway before the treadmill test to calculate their self-preferred walking speeds (distance/walking duration) [17]. Subsequently, the participants practiced the treadmill walking exercises until they became comfortable with their self-preferred speeds. We asked to walk naturally like in daily life on the treadmill during the test procedure. Subsequently, the participants rested for approximately 5 min. During the treadmill walking test, the participants were asked to walk for approximately 30–60 s at the onset of the treadmill walking exercise to maintain a stable walking pattern at their preferred walking speeds on the treadmill. Because the gait initiation and termination phases contained the acceleration and deceleration periods, which are not steady-state conditions, these phases were excluded from the gait analyses [7]. When a participant attained a stable walking pattern, an operator collected the treadmill walking data (one-minute periods). 

### 2.4. Data Analyses

**Figure 1:**
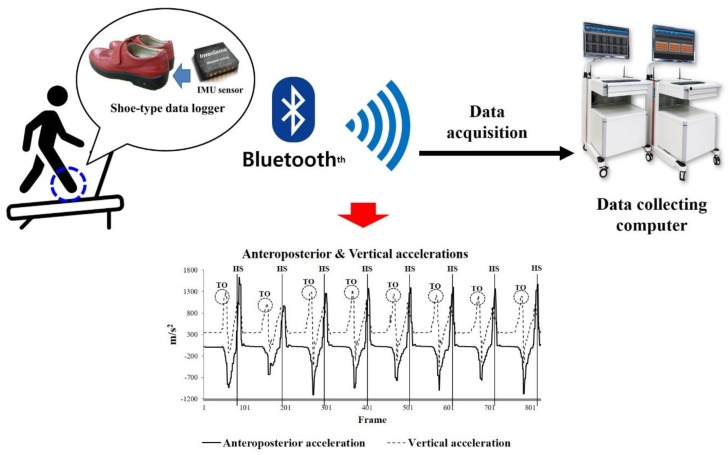
Shoe-type inertial measurement units (IMU) system and detection of gait events (HS is the Heel strike and TO is a Toe off).

The treadmill walking data were collected at 100 Hz and were filtered with a second-order Butterworth low-pass filter with a cut-off frequency of 10 Hz [31,32,33]. Linear acceleration was used to detect the gait events for heel strike (HS) and toe off (TO). HS is defined as the instance when the linear acceleration on the anteroposterior axis reaches its maximum positive (+) value. TO is defined as the instance when the linear acceleration on the vertical axis reaches its maximum positive (+) value during the gait cycle [31,33] (Figure 1).

Spatiotemporal parameters were calculated as follows: 1) Pace: walking speed, stride length, step length, and normalized variables, 2) Rhythm: cadence, and 3) Phases: single support, double support, and stance. Similar to the case of the GV, the percentage coefficient of variance (CV) values were also calculated for all the spatiotemporal parameters ((standard deviation/mean) × 100) [36,37]. 

To evaluate the bilateral coordination, we calculated the gait asymmetry (GA) and phase coordination index (PCI) based on a previous study [10]. The GA was the index for the temporal symmetry between the left and right feet during walking (see Equation (1)).

(1) $$\mathrm{Gait}\;\mathrm{Asymmetry}\;(\mathrm{GA})=100\times |\ln\;(\frac{\mathrm{short}\;\mathrm{swing}\;\mathrm{time}}{\mathrm{longswing}\;\mathrm{time}})|$$

The PCI value integrates the accuracy and consistency of the left–right movements during over-ground walking. In order to calculate the PCI value, we first calculated the mean of the swing time for the left and right legs, and the higher value of the average swing time was used as the reference for the opposing leg [10]. The relative step timing with respect to the stride time (360°), φi (180°), was determined as follows (Equation (2)).

(2) $$\varphi_{i}=360{}^{\circ}\times \frac{t_{\mathrm{Si}}-t_{\mathrm{Li}}}{t_{L\left(i+1\right)}-t_{\mathrm{Li}}}$$

In this case, t_Li_ and t_Si_ represent the times of the ith HS of the legs for long and short swing times, respectively, where t_L(I + 1)_ > t_Si_ > t_Li_. In order to maintain symmetry during walking, the φ_i_ value should be approximately 180°. Therefore, the mean value of the series of absolute differences (ABS) between φ_i_ and 180° (ABS_φ) was calculated to evaluate the overall accuracy. Furthermore, we also calculated the CV of φ in order to assess the level of consistency during the over-ground walking test. The PCI value was calculated as the combined CV of φ and percentage of ABS_φ (ABS_φ/180 × 100) [10]. All analyzed data shows in Supplementary Materials 2.

### 2.5. Statistical Analyses

All the statistical analyses were performed using a commercially available statistics software program for Windows (version 21.0, SPSS Inc. Chicago, IL). The Shapiro–Wilk test was used to determine whether the data were normally distributed. To examine which variables best defined the characteristics of healthy young adults, we used PCA with a varimax rotation. Prior to PCA, Z-normalization (value-mean/standard deviation) was performed for all the tested variables. The PC_s_ that had eigenvalues that exceeded unity were retained for interpretation. In addition, the Pearson’s product moment correlation analysis was used to determine the relationships between the PC_s_ and the PAs.

To establish the normative values for the gait-related variables, which includes mean and standard deviation as well as 95% of the confidence interval (CI), were also described for all participants. In addition, determining the gait characteristics for the healthy young adults, we conducted non-hierarchical cluster analyses (k-means method) to evaluate the essential gait ability according to the outcomes of the PC_1_ score. Prior to the non-hierarchical cluster analyses, hierarchical cluster analysis was conducted to determine the optimal number of clusters by means of a dendrogram [38], where the optimal number of clusters was between two and three. We concluded that three cluster groups were optimal for evaluating the gait ability of each group. The three cluster groups were defined as the top (top level), normal (middle level), and lowest (lowest level) groups based on the gait-related variables, according to the result of the PC_1_ score. One-way analysis of variance (ANOVA) with the Bonferroni correction was used to compare the differences among the cluster groups (top vs. middle vs. lowest) with respect to the pace, rhythm, phases, GV, and variables for the bilateral coordination. The statistical significance level for the post-hoc test was set to 0.0167 (0.05/3).

## 3. Results

### 3.1. PCA for Healthy Young Adults

**Figure 2:**
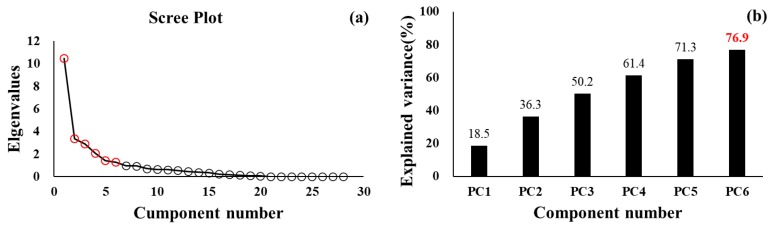
Principle component analysis (PCA) outcomes. (**a**) Cumulative percentage of the total variance (76.9%) explained by the principal components (PC_s_). (**b**) Scree plot of the 28 principal components.

A total of six PC_s_ indicated as a result of PCA, and PC_1_ to PC_6_ explained 76.9% of the total variance (Figure 2). The PC_s_ are interpreted as follows: 1) PC_1_ refers to the GV, which is composed of the CVs of the spatiotemporal parameters and φ, 2) PC_2_ refers to the pace parameters, and comprises the walking speed, stride, step length, and normalized variables, 3) PC_3_ refers to the phases and rhythm, and comprises the single support, double support, and stance phases, and cadence, 4) PC_4_ refers to the variables for bilateral coordination and GA, 5) PC_5_ refers to the demographic characteristics, and 6) PC_6_ refers to the PAs. The results of the factor loadings for the PC_s_ are listed in Table 2. In addition, the relationships between the PC_s_ and the total PAs indicated that PC_1_ exhibited a negative trend (r = −0.064, *P* < 0.05), whereas PC_2_ exhibited a positive trend (r = 0.063, *P* < 0.05) with respect to the frequency of the total PA.

### 3.2. Gait-Related Variables for All Participants

We established the normative values for the pace, rhythm, phases, GV, and variables for the bilateral coordination in Table 3 and Table 4. Table 3 and Table 4 exhibited the mean and standard deviation and 95% CI for the gait-related variables, according to all participants and each male and female.

### 3.3. Comparisons of the Gait Characteristics According to the Cluster Groups

**Figure 3:**
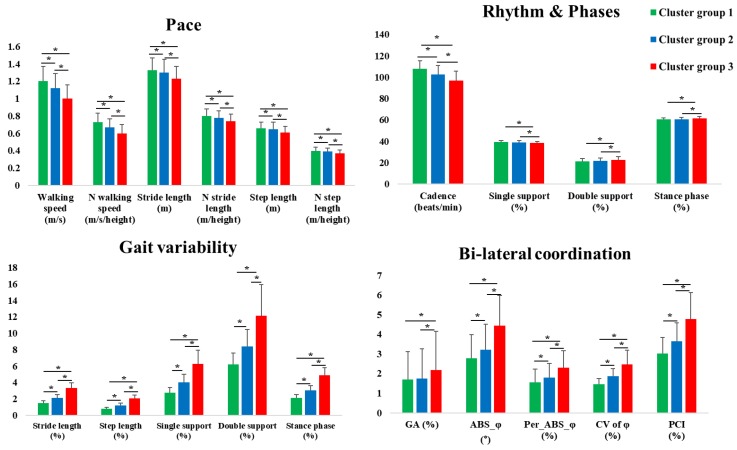
Results of pace, rhythm, phases, gait variability (GV), and bilateral coordination according to the cluster groups. The cluster groups are divided based on the stride length and stance phase. * denotes a significant difference. *P* < 0.0167.

We clustered group according to the PC_1_ score, which is the GV. There were no significant differences among the cluster groups 1 to 3. Figure 3 and Table S1 show the results of gait-related variables according to the cluster groups. Most variables indicated that there were significant differences among the cluster groups 1 to 3 regarding the pace (walking speed, stride, step length, and normalized variables), rhythm (cadence), GV (stride length, step length, single support phase, double support phase, and stance phase), and variables for bilateral coordination. In addition, there were significant differences between cluster group 1 and group 3 (top vs. lowest) as well as cluster group 2 and group 3 (middle vs. lowest) in the phases (single support phase, double support phase, and stance phase) and GA.

## 4. Discussion

The main findings of this study are as follows. 1) The contributions of the PC_s_ were 76.9% (PC_1_ to PC_6_), whereas PC_1_ (GV: 18.5%), PC_2_ (pace parameters: 17.8%), PC_3_ (phases and rhythm parameters: 13.9%), and PC_4_ (bilateral coordination and GA: 11.2%) were gait-related variables. 2) We established the normative values for both all participants and males and females. 3) All of the pace, rhythm, GV, and variables for bilateral coordination clearly classified the gait ability in the cluster groups. These findings are discussed in detail below.

Our study conducted PCA analyses in healthy young adults in which the PCs best defined their gait characteristics. These results indicated that PC_1_ to PC_6_ explained 76.9% of the observed variances. Our reported PC_1_ was the GV parameter, which explained 18.5% of the observed variables. It was composed of the GV for the pace and phase parameters, and the CV of φ among the PCI variables. In addition, PC_2_ was the pace parameter, which was composed of the stride length, step length, walking speed, and other normalized variables, and explained 17.8% of the observed variables. These variables have been used as the primary parameters of gait analyses [1,2,3,4,5,6]. The self-preferred speeds for walking and for the minimum individual energy cost may contribute to the maintenance of the minimum GV during gait [7,39]. Diminished walking speed and aging effects on the stride or step length during gait have also been reported [2]. In addition, the increased GV values may indicate possible decline in the neural control during walking [7,40] due to aging effects [7,8] and degenerative diseases [41], which may contribute to the decline of the dynamic stability during walking [2,3]. As indicated in previous studies, PAs may improve the gait stability [42,43,44,45]. Specifically, the increased lower limb strength enhances gait kinematics [46,47], which may decrease the GV during the walking cycle. As indicated, our results exhibited that PC_1_ (GV) and PC_2_ (Pace parameters) were related to the frequency of the PAs. In the cases where the participants performed PAs on a regular basis, their gait performances and postural stabilities were likely enhanced. In turn, this may indicate increased walking speeds or step lengths and decreased GV. Therefore, we propose the utilization of the GV and pace parameters to evaluate the gait ability.

In addition, our GV in the young adults indicated the stride length: 1.72% to 1.89%, step length: 1.01% to 1.09%, single support phase: 3.30% to 3.59%, double support phase: 7.24% to 7.62%, and stance phase: 2.55% to 2.72%. These results may be useful as the normative reference values because most previous studies focused on elderly adults ranging from 70 to 89 years old. Accordingly, these studies acknowledged that there was a lack of reference values for GV for young and middle-aged adults [2]. Our reported GV values for the young adults indicated relatively smaller outcomes compared to previous studies except for the double support phase. This finding may be associated with aging effects [7,8]. The elderly is weaker when compared with the young adults due to the loss of lower limb muscle strength, range of motion, improper muscle activation, and balance ability. These can be caused by the decline of the central motor control and automatic stepping mechanism, and would, thus, increase the GV [2,7].

Furthermore, our results indicated the GA and PCI values ranged between 1.72%–1.78% and 3.23%–3.48%, respectively. Our reported values for the GA and PCI exhibited narrow ranges when compared to previous studies [10,11,12]. In previous studies, the ranges of GA and PCI in the young adults ranged from 0.84%–1.26% and 2.47%–5.40%, respectively [10,11,12]. These results may be related to those evoked based on relatively larger sample sizes and numerous continuous steps in treadmill walking. Thus, they may be influenced to increase data consistency for the GA and PCI variables. The GA and PCI are variables used for evaluating the bilateral coordination that integrates the accuracy and consistency of the left-right movement when walking. Increased GA and PCI values imply more reduced bilateral coordination that may be related to a worsened gait coordination capacity and worsened dynamic stability [10,11,12,13]. Therefore, we suggest that our results may provide more precise and reliable data to understand the characteristics of healthy young adults as reference values. 

We clustered the group according to the PC1 score, which is the GV. The pace, rhythm, GV, and variables for bilateral coordination classified the gait performance in the cluster groups. Specifically, cluster group 1 (top level) yielded faster walking speed and larger stride length compared to cluster groups 2 (middle level) and 3 (lowest level). We determined that the gait performance trends were worse for cluster group 3. Our participants who had shortened stride lengths and longer stance phases yielded slower walking speeds and larger GV, GA, and bilateral coordination. Increased walking speeds resulted in increases in the single support phase, whereas decreased walking speeds resulted in increases of the double support and stance phases [48]. The slower walking speed led to increases of the bilateral coordination because this condition was not commonly experienced in daily lives, and, thus, required more attention and dynamic stability [11]. Specifically, shortened stride lengths reflected weakened muscle strengths, which could influence the alterations to lower limb kinematics and kinetics [4]. Similar gait patterns were commonly observed in elderly adults. As shown in previous studies, increased step widths and double support phases were observed in the cases of elderly adults to increase the dynamic stability when walking. These changes evoked longer stance phases in response to the reduced lower limb strengths [1,2,4]. 

In addition, cluster group 3 (lowest level) yielded similar GV and PCI ranges compared with the healthy elderly population evaluated in previous studies (our young adults vs. elderly adults in previous studies = stride length: 3.31% vs. 2.23%–2.42%, single support phase: 6.24% vs. 3.90%–6.00%, double support phase: 12.16% vs. 6.00%–6.80%, and PCI: 4.11% vs. 3.30% to 6.10%) [1,6,10,13,49]. Increased PCI values imply poorer bilateral coordination that may be related to worsened gait coordination capacity and worsened dynamic stability [10,11,12,13]. These findings may be related to the slower walking speeds that required increased dynamic stability achieved by the activated muscles. These findings may also reflect the increased time spent in the single limb stance and the increased medio-lateral displacement of the center of mass [9]. These results may reflect inadequate muscular strengths and ranges of motion for the hip and ankle joints during gait, such as the inability to induce enough ankle power during the toe-off phase [2,4,7]. In our study, the GV and bilateral coordination indicated a negative correlation with these total METs during PAs and frequencies (see Table S1), which may be similar results with the previous studies that decrease the dynamic stability by reduced muscle strength [2,4,9]. However, we did not consider objective measurements of muscular strengths or more complex variables during gait analyses, such as the joint torque, which may provide insights into the gait characteristics, according to the gait performances. Even though this may not be valid in the case of young adults, worse GV and bilateral coordination outcomes may be indicative of a decline in gait performance [7,10,40]. Instead, the worsened gait performance may cause an increased risk of motor function decline that is ultimately related to the ability to control the gait patterns [2]. Therefore, the participants who yielded poor GV and bilateral coordination outcomes may need to enroll in intervention programs to enhance their motor functions. 

There are several strengths associated with this study. First, we analyzed relatively large samples of healthy young adults and determined the gait characteristics using various methods that included establishing reference values, which is compared to the gait performances. Second, our gait-related variables are considered reliable. For instance, our results for the GA and PCI exhibited relatively narrow ranges compared to previous studies [10,11,12]. These results may be related to those evoked based on relatively larger sample sizes and numerous continuous steps in treadmill walking. Previous studies recommended that the collection of 40 consecutive steps [9] is required to enhance data accuracy. Our data were based on continuous treadmill walking during one-minute intervals and may enhance the data accuracy and consistency of gait analyses. Alternative methods may exist to emulate the actual walking patterns of the participants. 

We also recognize the limitations associated with this study. First, we recruited participants with prior experience in using the treadmill. The participants were asked to walk naturally on the treadmill, as in daily life, to the greatest extent possible during the testing procedure. Some previous studies have reported no significant differences in the spatiotemporal parameters between treadmill walking and over-ground walking in healthy humans [17,50,51,52]. However, treadmill walking may reduce the variance of the step when compared with over-ground walking because the treadmill mechanically regulates the walking speed and constrains the participants to walk along a straight line [17]. Thus, it may stimulate increased cadence and shorter strides [53,54]. Nevertheless, analyzing the gait characteristics in healthy young adults may be meaningful because several studies have reported the positive effects of gait training programs using treadmills [18,19,20,21,22,23,24,25,26]. Second, there were limitations in the direct comparisons of GV with previous studies because most previous studies focused on elderly adults (who ranged in age from 70 to 89 years). Thus, it is necessary to establish the GV values according to all ages (e.g., aged 20 to over 80). In addition, Al-Obaidi et al. [11] and Oberg et al. [5] presented reference values at various conditions, such as a slower, preferred, and faster speeds. Almarwani et al. [7] suggested the conduct of gait analyses at various walking speed conditions because challenge conditions (slower or faster speed) may be useful for evaluating the motor functions and the determination of the decline of gait function. Thus, future studies will consider various walking conditions, such as slower or faster gait speeds. Evoked outcomes may be used for advanced gait analyses. Furthermore, all participants wore shoe-type data loggers of size selected by them to fit their feet. However, the shoe-type data loggers are not usually worn in daily life. Therefore, it was possible that they may have affected the walking patterns of the participants. Lastly, we did not compare the gait characteristics according to sex. Thus, future studies need to consider the essential gait characteristics at the various walking conditions, which may be meaningful to understand the gait characteristics depending on the sex.

## 5. Conclusions

Our study investigated the gait characteristics in healthy young adults ranging in age from 19 to 30 years old using shoe-type inertial measurement units (IMU) during treadmill walking (one-minute intervals). We determined the characteristics of healthy young adults using PCA. It may be meaningful to utilize the pace, rhythm, and phase parameters as well as the GV values to evaluate the gait performance. In addition, we established the normative values of both all participants and males and females. Lastly, the pace, rhythm, GV, and variables for bilateral coordination classified the gait ability in young adults. Specifically, the participants who attained shortened stride lengths and longer stance phases yielded worse GV and bilateral coordination outcomes and needed to enroll in interventional programs to enhance their motor functions and reduce the potential risk that would lead to the decline of their gait abilities. Therefore, we consider that our results are meaningful and aid the understanding of gait patterns in healthy adults. We consider that they could be proven useful as reference values for assessing gait analyses in the future.

## Figures and Tables

**Table 1:** Demographic characteristics.

	All Participants N = 1478	Male N = 558	Female N = 920	*P*-Value *
Age (years)	21.70 ± 2.37	22.48 ± 2.41	21.23 ± 2.22	<0.001
Height (cm)	165.84 ± 8.40	174.17 ± 5.66	160.79 ± 5.12	<0.001
Body weight (kg)	63.06 ± 13.07	74.20 ± 11.75	56.30 ± 8.36	<0.001
Body mass index (kg/m^2^)	22.75 ± 3.30	24.41 ± 3.30	21.75 ± 2.87	<0.001
Waist circumference (cm)	66.84 ± 7.87	72.96 ± 6.69	63.12 ± 5.98	<0.001
Blood pressure (mmHg)	116.06 ± 13.06	123.74 ± 12.04	111.41 ± 11.35	<0.001
Total PA (frequency/week)	7.91 ± 4.26	9.26 ± 4.39	7.10 ± 3.96	<0.001
Total METs for PA (METs/min)	1923.99 ± 3899.35	2533.86 ± 3588.24	1554.10 ± 4033.68	<0.001

Mean ± standard deviation. BMI: Body mass index. PA: physical activity. MET: metabolic equivalents. * is a significant difference between males and females.

**Table 2:** Factor loading outcomes for principal components.

Variables		Components					
		PC_1_	PC_2_	PC_3_	PC_4_	PC_5_	PC_6_
**Gait variability**	CV of step length	**0.895**	−0.149	−0.247	0.115	−0.057	−0.031
	CV of stance phase	**0.886**	−0.197	−0.268	0.124	−0.058	−0.029
	CV of stride length	**0.882**	−0.171	−0.100	0.099	−0.075	−0.044
	CV of single support	**0.810**	−0.217	−0.356	0.166	−0.056	−0.047
	CV of double support	**0.764**	−0.066	0.055	0.082	−0.073	−0.015
	CV of φ	**0.698**	−0.296	−0.123	0.314	−0.097	−0.075
**Pace**	N step length	−0.205	**0.928**	0.244	−0.074	−0.042	0.001
	N stride length	−0.207	**0.927**	0.250	−0.071	−0.040	0.002
	Step length	−0.162	**0.894**	0.234	−0.080	0.280	0.060
	Stride length	−0.162	**0.894**	0.234	−0.080	0.281	0.060
	Walking speed	−0.343	**0.677**	0.553	−0.100	0.181	0.031
	N walking speed	−0.374	**0.653**	0.568	−0.092	−0.054	−0.012
**Phases** **and** **rhythm**	Double support	0.124	−0.396	**−0.872**	0.074	0.018	−0.025
	Single support	−0.114	0.366	**0.832**	−0.109	0.002	0.009
	Stance phase	0.121	−0.386	**−0.822**	0.032	0.037	−0.038
	Cadence	−0.441	0.041	**0.738**	−0.085	−0.045	−0.028
**Bilateral** **coordination** **and** **GA**	Percentage_ABS_φ	0.275	−0.143	−0.049	**0.898**	−0.032	−0.022
	ABS_φ	0.275	−0.143	−0.049	**0.898**	−0.032	−0.022
	PCI	0.512	−0.236	−0.091	**0.767**	−0.067	−0.050
	Φ	0.094	−0.044	0.042	**−0.675**	0.024	−0.034
	GA	0.060	0.003	−0.045	**0.490**	0.028	−0.027
**Demographic characteristics**	Waist circumference	−0.035	0.080	−0.062	−0.018	**0.839**	0.090
	Sex	−0.005	−0.068	−0.074	0.043	**−0.815**	−0.085
	BMI	−0.116	0.056	−0.159	0.002	**0.738**	0.046
	Blood pressure	0.014	−0.009	0.011	−0.012	**0.691**	−0.031
	Age	−0.104	0.073	0.054	0.004	**0.298**	0.112
**Physical activities**	Total METs for PA	−0.048	−0.004	−0.018	−0.023	0.092	**0.887**
	Total PA frequency	−0.064	0.063	0.041	−0.030	0.202	**0.848**
	**Explained Variance (%)**	18.5	17.8	13.9	11.2	9.9	5.6

ABS: absolute. BMI: body mass index. CV: coefficient of variance. GA: gait asymmetry. METs: metabolic equivalents. N: normalized. PA: physical activities. PC: principal component. PCI: phase co-ordinate index.

**Table 3:** Results of gait-related variables in healthy young adults: spatiotemporal parameters and gait variability (GV).

Variables	All Participants (N = 1478) (min to max, 95% CI)	Male (N = 558) (min to max, 95% CI)	Female (N = 920) (min to max, 95% CI)
Walking speed (m/s)	1.16 ± 0.18 (1.15 to 1.17)	1.22 ± 0.18 (1.20 to 1.23)	1.12 ± 0.17 (1.11 to 1.13)
N walking speed (m/s/height)	0.70 ± 0.10 (0.69 to 0.70)	0.70 ± 0.10 (0.69 to 0.71	0.70 ± 0.11 (0.69 to 0.71)
Stride length (m)	1.31 ± 0.15 (1.30 to 1.32)	1.38 ± 0.14 (1.37 to 1.39)	1.27 ± 0.14 (1.26 to 1.28)
N stride length (m/height)	0.79 ± 0.08 (0.79 to 0.79)	0.79 ± 0.08 (0.79 to 0.80)	0.79 ± 0.08 (0.78 to 0.79)
Step length (m)	0.66 ± 0.07 (0.65 to 0.66)	0.69 ± 0.07 (0.68 to 0.70)	0.63 ± 0.07 (0.63 to 0.64)
N step length (m/height)	0.39 ± 0.04 (0.39 to 0.40)	0.40 ± 0.04 (0.39 to 0.40)	0.39 ± 0.04 (0.39 to 0.40)
Cadence (beats/min)	105.23 ± 8.62 (104.79 to 105.67)	105.15 ± 7.97 (104.49 to 105.81)	105.28 ± 8.99 (104.70 to 105.86)
Single support phase (%)	39.21 ± 1.44 (39.14 to 39.28)	39.35 ± 1.46 (39.23 to 39.47)	39.13 ± 1.43 (39.03 to 39.22)
Double support phase (%)	21.56 ± 2.74 (21.42 to 21.70)	21.33 ± 2.64 (21.11 to 21.55)	21.70 ± 2.80 (21.52 to 21.88)
Stance phase (%)	60.77 ± 1.45 (60.70 to 60.85)	60.68 ± 1.35 (60.57 to 60.79)	60.83 ± 1.51 (60.73 to 60.93)
CV of stride length (%)	1.84 ± 0.65 (1.80 to 1.87)	1.76 ± 0.63 (1.70 to 1.81)	1.89 ± 0.67 (1.85 to 1.93)
CV of step length (%)	1.06 ± 0.43 (1.04 to 1.08)	1.01 ± 0.41 (0.97 to 1.05)	1.09 ± 0.44 (1.06 to 1.12)
CV of single support phase (%)	3.48 ± 1.37 (3.41 to 3.55)	3.30 ± 1.23 (3.20 to 3.40)	3.59 ± 1.44 (3.50 to 3.68)
CV of double support phase (%)	7.47 ± 2.64 (7.34 to 7.61)	7.24 ± 2.70 (7.01 to 7.46)	7.62 ± 2.59 (7.45 to 7.78)
CV of stance phase (%)	2.66 ± 0.98 (2.61 to 2.71)	2.55 ± 0.93 (2.47 to 2.63)	2.72 ± 1.01 (2.66 to 2.79)

Mean ± standard deviation. N: normalized. CI: Confidence interval. CV: Coefficient of variance.

**Table 4:** Results of gait-related variables in healthy young adults: gait asymmetry (GA) and phase coordination index (PCI) variables.

Variables	All Participants (N = 1478) (min to max, 95% CI)	Male (N = 558) (min to max, 95% CI)	Female (N = 920) (min to max, 95% CI)
GA (%)	1.76 ± 1.51 (1.68 to 1.83)	1.72 ± 1.62 (1.59 to 1.86)	1.78 ± 1.45 (1.68 to 1.87)
φ (°)	178.80 ± 2.23 (178.69 to 178.91)	178.88 ± 2.26 (178.69 to 179.07)	178.75 ± 2.21 (178.61 to 178.89)
ABS_φ (°)	3.06 ± 1.32 (2.99 to 3.12)	2.94 ± 1.32 (2.83 to 3.05)	3.13 ± 1.32 (3.04 to 3.21)
Percentage_ABS_φ (%)	1.70 ± 0.73 (1.66 to 1.74)	1.63 ± 0.73 (1.57 to 1.69)	1.74 ± 0.73 (1.69 to 1.79)
CV of φ (%)	1.69 ± 0.49 (1.66 to 1.71)	1.59 ± 0.46 (1.56 to 1.63)	1.74 ± 0.49 (1.71 to 1.78)
PCI (%)	3.39 ± 1.06 (3.33 to 3.44)	3.23 ± 1.00 (3.14 to 3.31)	3.48 ± 1.08 (3.41 to 3.55)

Mean ± standard deviation. ABS: Absolute. CI: Confidence interval. CV: Coefficient of variance. GA: Gait asymmetry. PCI: Phase coordinate index.

## Data Availability

The datasets generated and/or analyzed during the current study are not publicly available due to intellectual property reasons, but these are available upon a reasonable request.

## Supplementary Materials

The following are available online at https://www.mdpi.com/1424-8220/20/7/2095/s1, Table S1. Association of physical activity with GV and bilateral coordination. Supplementary Materials 1: IRB number. Supplementary Materials 2: Raw data.
