# Supplementary material for: Gait Characteristics Based on Shoe-Type Inertial Measurement Units in Healthy Young Adults during Treadmill Walking
Source: Sensors (Basel). 2020 Apr 8;20(7):2095. doi: 10.3390/s20072095 (PMC7180462; doi:10.3390/s20072095)
Supplement: Supplementary file 1 [file sensors-20-02095-s001.zip › Table S1.docx]

| **Table A1. Association of physical activity with GV and bi-lateral co-ordination** | | | | | | | | | | |
| --- | --- | --- | --- | --- | --- | --- | --- | --- | --- | --- |
|  | CV of stride length  (%) | CV of step length  (%) | CV of single support phase  (%) | CV of double support phase  (%) | CV of stance phase  (%) | φ  (°) | ABS_φ  (°) | Percentage_ABS_φ  (%) | CV of φ  (%) | PCI  (%) |
| Total PA (frequency/week) | **-0.118^**^** | **-0.115^**^** | **-0.133^**^** | **-0.108^**^** | **-0.121^**^** | 0.011 | **-0.078^**^** | **-0.078^**^** | **-0.146^**^** | **-0.121^**^** |
| Total METs for PA (METs/min) | **-0.092^**^** | **-0.080^**^** | **-0.080^**^** | -0.047 | **-0.073^**^** | -0.008 | **-0.056^*^** | **-0.057^*^** | **-0.098^**^** | **-0.084^**^** |
| ABS: Absolute, CV: Coefficient of variance, MET: metabolic equivalents, PA: physical activity, PCI: Phase coordinate index, * P < 0.05, ** P < 0.01. | | | | | | | | | | |
